# Interventions to reduce preterm birth and stillbirth, and improve outcomes for babies born preterm in low- and middle-income countries: A systematic review

**DOI:** 10.7189/jogh.11.04050

**Published:** 2021-12-30

**Authors:** Elizabeth Wastnedge, Donald Waters, Sarah R Murray, Brian McGowan, Effie Chipeta, Alinane Linda Nyondo-Mipando, Luis Gadama, Gladys Gadama, Martha Masamba, Monica Malata, Frank Taulo, Queen Dube, Kondwani Kawaza, Patricia Munthali Khomani, Sonia Whyte, Mia Crampin, Bridget Freyne, Jane E Norman, Rebecca M Reynolds, DIPLOMATIC Collaboration

**Affiliations:** 1 Medical Research Council Centre for Reproductive Health, University of Edinburgh, Queen’s Medical Research Institute, Edinburgh, UK; 2 Centre for Reproductive Health, College of Medicine, University of Malawi, Blantyre, Malawi; 3 Department of Health Systems & Policy, School of Public Health and Family Medicine, College of Medicine, University of Malawi, Blantyre, Malawi; 4 Department of Obstetrics & Gynaecology, College of Medicine, University of Malawi, Blantyre, Malawi; 5 Department of Paediatrics, College of Medicine, University of Malawi, Blantyre, Malawi; 6 Malawi-Liverpool Wellcome Trust Research Program, Blantyre, Malawi; 7 Malawi Epidemiology and Intervention Research Unit, Lilongwe, Malawi; 8 Institute of Infection & Global Health, University of Liverpool, Liverpool, UK; 9 Faculty of Health Sciences, University of Bristol, Bristol, UK; 10 Centre for Cardiovascular Science, University of Edinburgh, Queen’s Medical Research Institute, Edinburgh, UK

## Abstract

**Background:**

Reducing preterm birth and stillbirth and improving outcomes for babies born too soon is essential to reduce under-5 mortality globally. In the context of a rapidly evolving evidence base and problems with extrapolating efficacy data from high- to low-income settings, an assessment of the evidence for maternal and newborn interventions specific to low- and middle-income countries (LMICs) is required.

**Methods:**

A systematic review of the literature was done. We included all studies performed in LMICs since the Every Newborn Action Plan, between 2013 - 2018, which reported on interventions where the outcome assessed was reduction in preterm birth or stillbirth incidence and/or a reduction in preterm infant neonatal mortality. Evidence was categorised according to maternal or neonatal intervention groups and a narrative synthesis conducted.

**Results:**

179 studies (147 primary evidence studies and 32 systematic reviews) were identified in 82 LMICs. 81 studies reported on maternal interventions and 98 reported on neonatal interventions. Interventions in pregnant mothers which resulted in significant reductions in preterm birth and stillbirth were (i) multiple micronutrient supplementation and (ii) enhanced quality of antenatal care. Routine antenatal ultrasound in LMICs increased identification of fetal antenatal conditions but did not reduce stillbirth or preterm birth due to the absence of services to manage these diagnoses. Interventions in pre-term neonates which improved their survival included (i) feeding support including probiotics and (ii) thermal regulation. Improved provision of neonatal resuscitation did not improve pre-term mortality rates, highlighting the importance of post-resuscitation care. Community mobilisation, for example through community education packages, was found to be an effective way of delivering interventions.

**Conclusions:**

Evidence supports the implementation of several low-cost interventions with the potential to deliver reductions in preterm birth and stillbirth and improve outcomes for preterm babies in LMICs. These, however, must be complemented by overall health systems strengthening to be effective. Quality improvement methodology and learning health systems approaches can provide important means of understanding and tackling implementation challenges within local contexts. Further pragmatic efficacy trials of interventions in LMICs are essential, particularly for interventions not previously tested in these contexts.

Despite major global improvements in maternal and neonatal health during the Millennium Development Goals era, unacceptably high levels of preventable morbidity and mortality remain in many areas of the world [1]. Reductions in neonatal mortality (deaths within the first 28 days of life) have lagged behind those of overall mortality in children under 5 years of age, and as a result neonatal mortality accounts for 45% of total under-5 mortality worldwide [2]. Prematurity and associated complications are now the most frequent cause of death in all children younger than 5. Improvements in newborn survival have been slower in sub-Saharan Africa with current trends suggesting it will take over a century to achieve rates of newborn survival comparable to North America or Europe [3]. Sub-Saharan Africa has the highest stillbirth rates of any region, an under-recognised and neglected global public health issue responsible for 2.6 million third trimester fetal deaths worldwide in 2015 [4].

Despite evidence suggesting that 71% of neonatal deaths could be averted through scale-up of existing interventions, interventional studies in low-resource settings are limited [5]. This is reflected in the WHO guidelines on maternal and newborn care, which are derived primarily from studies done in high-income settings [6-8]. Extrapolation of efficacy estimates between settings is problematic as seen in the Antenatal Corticosteroids Trial (ACT) [9] and Fluid Expansion As Supportive Therapy (FEAST) trials [10,11]. There is therefore a major requirement for further efficacy studies of many interventions in low- and middle-income countries. In addition to this, there is an ongoing need for dissemination of current research findings in these settings in order to target future research appropriately.

This review presents the current evidence from studies done in LMICS over the last five years. We included studies or systematic reviews which reported on interventions targeted at (i) the prevention of preterm birth and stillbirth and (ii) decreasing mortality in preterm and low birth weight infant and ill newborns. This review aims to update existing guidance from the Every Newborn Action Plan [6-8] and report on the latest evidence from recent studies.

## METHODS

A systematic literature review was done for studies published between (January 2013-May 2018) which reported on interventions in both mothers and infants from LMICs with the outcomes of 1) reducing preterm birth incidence 2) reducing stillbirth incidence 3) reducing neonatal mortality in preterm, low-birth weight, or unwell infants. The review adhered to MOOSE and PRISMA guidelines for reporting of systematic reviews and was pre-registered with Prospero (CRD42018099173). Key outcomes were defined as following: stillbirth was defined as baby born without signs of life after 28 weeks gestation; preterm birth was defined as Baby born before 36 weeks gestation; neonatal mortality was defined as death of baby born live, before 28 days of age [12].

MEDLINE, EMBASE, Web of Science, LILACS, CINAHL, Global health, MIDIRS, and the Cochrane Library were searched using keywords related to the above outcomes and limited to LMICs as defined by World Bank groupings [13]. The search was time-limited in order to build on previous reviews published in 2014 as part of the Lancet Every Newborn Series [5]. The keyword searches were done separately for maternal and neonatal interventions. Selected studies were screened by two reviewers (EW, DW). Inclusion criteria were: intervention studies published 2013-2018 in pregnant women or preterm newborns reporting on outcomes of preterm birth, stillbirth or neonatal mortality conducted in LMIC (or systematic reviews reporting >50% LMIC data or providing LMIC subgroup analysis. Accepted study designs were RCTs, cohort studies, case/control studies or before/after studies based in either community or hospital settings on both singleton and multiple pregnancies. Studies were excluded if they did not adequately report sample size calculation, or if they reported on wider public health interventions not exclusively aimed at pregnant women. Editorials, commentaries, reviews, conference abstracts and trial protocols were excluded, as were studies not published in the English language.

**Figure 1:**
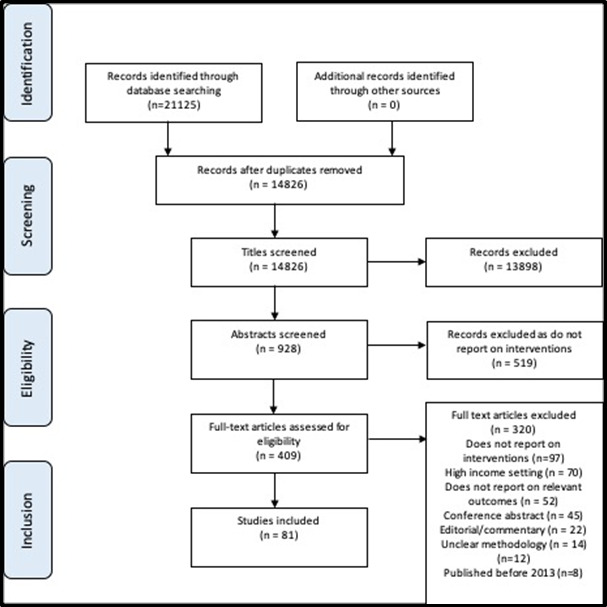
PRISMA flow diagram for maternal intervention study selection.

**Figure 2:**
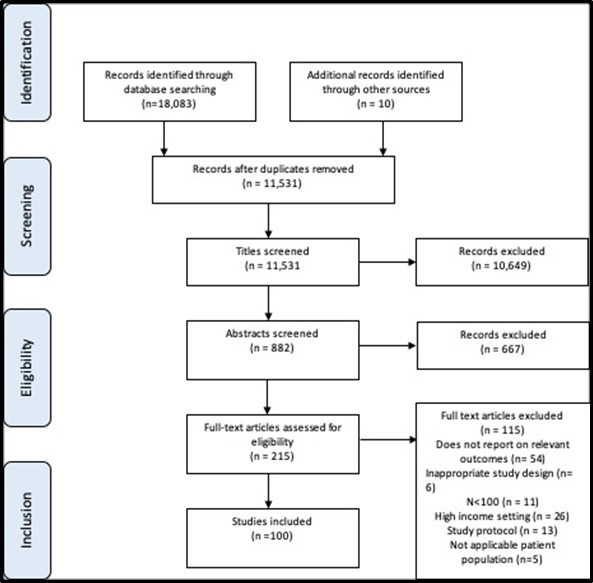
PRISMA flow diagram for neonatal intervention study selection.

Reference lists from relevant articles were also searched. Details of the search strategy can be found in Table S1 in the **Online Supplementary Document**. The PRISMA flow diagrams of search strategies for maternal interventions and neonatal interventions can be seen in **Figure 1** and **Figure 2****.**

In light of the results from the ACT trial showing potential harm from antenatal corticosteroids in LMICs, and the pending publication of the WHO ACTION trials [14], we excluded studies related to antenatal corticosteroids and preterm birth prevention [15].

### Evidence synthesis

All studies which met these criteria were entered into an evidence gap map which is a visual representation of the studies in each area, demonstrating the volume of evidence in each area [16]. antenatal and delivery care (**Table 1**), lifestyle interventions (**Table 2**), health systems, training and guidelines (**Table 3**), pharmacological interventions (**Table 4**), nutritional supplements (**Table 5**), and community groups (**Table 6**). Neonatal interventional studies were grouped as: infection prevention and treatment (**Table 7**), respiratory support (**Table 8**), cardiovascular support (**Table 9**), health systems, training and guidelines (**Table 10)**, feeding and nutrition (**Table 11**) and community mobilization (**Table 12**). Complex interventions were disaggregated where possible and the relevant data for each of the above groups was assessed independently.

**Table 1:** Maternal interventions – characteristics of individual studies (antenatal and delivery care)

Authors	Year of publication	Dates of study	Location	Study type	Study setting	Population
Afulani. [17]	2016	2007	Ghana	Retrospective cohort	Population level	4868 women from DHS who had given birth in previous 5 y
Amoakoh-Coleman et al. [18]	2016	December 2013-May 2014	Ghana	Pre-post implementation	11 health facilities	926 pregnant women
Beauclair et al. [23]	2014	1 April 2006-31 March 2009	South Africa	Retrospective cohort	Public perinatal clinic	34 671 pregnant women (only singletons
Biswas et al. [24]	2018	2016	Bangladesh	Prospective cohort	Community	450 pregnant women including 72 complicated mothers
Chinkhumba et al. [87]	2014	January-August 2013	Tanzania, Malawi, Burkina Faso, Uganda, DRC, Senegal, Guinea Bissau	Systematic literature review and meta-analysis	Health facility	9 studies covering 47 475 women
Day et al. [88]	2016	1 January 2009-31 December 2015	Bangladesh	Cross-sectional	Referral hospital (records review)	23 986 singleton term deliveries
Ganchimeg et al. [89]	2016	1 May 2010-31 December 2011	29 LMICs	Secondary analysis of the WHO multicountry survey on MCH	N/A	29 647 pregnant women with previous Caesarean-section and no current complications
Godlonton and Okeke [90]	2016	2010	Malawi	Retrospective cohort	Population level	9339 pre-ban and 10 341 post-ban
Goudar et al. [91]	2015	January 2010-December 2013	India	Retrospective cohort	2 health facilities in Belgaum and Nagpur	107 884 deliveries
Goldenberg et al. [30]	2018	July 2014-May 2016	DRC, Guatemala, Kenya, Pakistan and Zambia	Cluster randomised controlled trial	Health centre	28 263 intervention and 23 160 control
Kayiga et al. [92]	2018	November 2015-May 2016	Uganda	Prospective cohort	Tertiary referral hospital	1425 women with PROM
Khanam et al. [93]	2018	2011-2013	Bangladesh	Prospective cohort	Population level	24 271 deliveries
Lassi et al. [25]	2016	January 2015	LMICs	Systematic literature review and meta-analysis	Community	N/A
Lee et al. [31]	2017	January 2012-March 2016	Bangladesh	Cluster randomised controlled trial	Antenatal care	3818 intervention and 3557 pregnant women GA 13-19w
Mbuagbaw et al. [26]	2016	June 2015	LMICs	Cochrane review	Community	34 trials (NB 5 of these in HICs)
Mbuyita et al. [27]	2015	unspecified	Tanzania	Pre-post implementation	10 antenatal clinics	257 pregnant women
McDiehl et al. [20]	2017	2015	Uganda	Prospective cohort	Regional referral hospital	4231 women presenting for delivery
Muhindo et al. [71]	2016	June-October 2014 and December 2014-February 2015	Uganda	Prospective cohort	Community	289 women 12-20 weeks gestation
Nimi et al. [28]	2016	December 2012-February 2013	Angola	Cross-sectional	Tertiary referral hospital	995 women delivering in hospital (interviews)
Asundep et al. [19]	2014	July-November 2011	Ghana	Cross-sectional	2 public hospitals and 16 TBAs	629 women presenting for delivery
Orobaton et al. [72]	2016	April-November 2015	Nigeria	Prospective cohort	Community	9427 pregnant women
Roh et al. [73]	2017	December 2014-October 2015	Uganda	Case-control	Community	380 control vs 185 intervention HIV+ve pregnant women
Salam et al. [76]	2014	May 2013	22 LMICs	Systematic literature review and meta-analysis	Community	32 studies
Scott et al. [74]	2018	November 2013- November 2015	The Gambia, Burkino Faso and Benin	Cluster randomised controlled trial	Community	4731 pregnant women

**Table 2:** Maternal interventions- characteristics of individual studies (lifestyle)

Authors	Year of publication	Dates of study	Location	Study type	Study setting	Population
Alexander et al. [70]	2018	June 2013-October 2015	Nigeria	Randomised control trial	Community	324 pregnant women (162 intervention and 162 control)
Wang et al. [68]	2017	December 2014-July 2016	China	Randomised control trial	Antenatal care	300 singleton women at GA 10 weeks with BMI>24
Wang et al. [69]	2015	20 June-30 November 2013	China	Retrospective cohort	Antenatal care	2750 pregnant women with GDM. 74.9% underwent intervention

**Table 3:** Maternal interventions- characteristics of individual studies (health systems, training and guidelines)

Authors	Year of publication	Dates of study		Location	Study type	Study setting	Population
Amoakoh-Coleman et al. [18]	2016	December 2013-May 2014		Ghana	Pre-post implementation	11 health facilities	926 pregnant women
Asare et al. [80]	2017	January 2014- May 2016		Ghana	Pre-post implementation	Teaching hospital	Women with SCD 158 pre and 90 post- intervention
Ballard et al. [22]	2016	May- December 2014		Ethiopia	Cross-sectional (questionnaires of women who delivered in the past 12 mo)	Community	4442 women who had delivered in previous 12 mo (randomly selected)
Byaruhanga et al. [81]	2015	July 2012- December 2013		Uganda	Prospective equally randomised clinical trial	Teaching hospital	1971 women in active labour
Chomba et al. [85]	2017	March 2005-February 2007		DRC, Guatemala, Kenya, Pakistan and Zambia	Prospective cohort	Community	22 745 controls and 35 074 intervention
Gomez et al. [86]	2018	March 2014-February 2017		Ghana	Cluster randomised controlled trial	40 hospitals	67 659 births post-intervention +38192 births pre-intervention
Goudar et al. [91]	2015	January 2010- December 2013		India	Retrospective cohort	2 health facilities in Belgaum and Nagpur	107884 deliveries
Maaløe et al. [77]	2017	1 October 2014- 31 January 2015		Tanzania	Pre-post implementation	Referral hospital	All labouring women in the hospital- baseline = 3690 intervention = 3087
Mgaya et al. [78]	2016	October 2013-March 2014 then July2015- November 2015	Tanzania		Pre-post implementation	National referral hospital	260 deliveries pre and 250 post
Okonofua et al. [79]	2013	Baseline Jan-May 2008, April-June 2009		Nigeria	Pre-post implementation	6 teaching hospitals	219 women with eclampsia
Pasha et al. [61]	2013	March 2009-30 September 2011		Pakistan, Kenya, Zambia, Guatemala and Argentina	Cluster randomised controlled trial	Community	55 712 intervention and 54 822 control over 106 clusters
Patel et al. [218]	2017	1 July 2012- 30 November 2013		India	Prospective cohort	Community	7050 pregnant women
Srofenyoh et al. [83]	2013	2007-2009		Ghana	Quality improvement	Regional referral hospital	All women delivering in the facility

**Table 4:** Maternal interventions- characteristics of individual studies (Pharmacological interventions)

Authors	Year of publication	Dates of study	Location	Study type	Study setting	Population
Bellad et al. [32]	2018	October 2013 –July 2015	India	Randomised placebo-controlled trial	Antenatal care	1727 women GA 13-20 weeks with vaginal pH≥5
Brizot et al. [98]	2015	1 June 2007-31 October 2013	Brazil	Randomised placebo-controlled double-blinded	Single hospital	390 twin pregnancies 18-22w GA
Chagomerana et al. [94]	2017	April 2012-November 2015	Malawi	Retrospective cohort	Regional referral hospital	3074 HIV infected pregnant women delivering at >27 weeks gestation
Cluver et al. [218]	2018	January 2016-April 2017	South Africa	Randomised control trial	Hospital	Women with pre-eclampsia 26-37w GA. 59 intervention and 60 placebo
Day et al. [88]	2016	1 January 2009-31 December 2015	Bangladesh	Cross-sectional	Referral hospital (records review)	23 986 singleton term deliveries
Gupta et al. [33]	2013	October 2005 – March 2007	India	Randomised control trial	Referral hospital	242 women GA 12-24w with abnormal vaginal flora
Haghighi et al. [99]	2017	December 2001-November 2012	South Africa	Randomised control trial	Teaching hospital	315 women with threatened pre-term labour (progesterone 159, nifedipine 156)
Jiang et al. [100]	2016	November 2013-July 2015	China	Randomised control trial	County level hospital	Pregnant women <20w GA 232 intervention 234 control
Lancaster et al. [96]	2016	12 March 2001-6 January 2010	Uganda and Zimbabwe	Prospective cohort	Community	160 HIV infected pregnant women
Lee et al. [31]	2017	January 2012-March 2016	Bangladesh	Cluster randomised controlled trial	Antenatal care	3818 intervention and 3557 pregnant women GA 13-19w
Li et al. [34]	2017	2001-2015	China	Prospective cohort with effect estimates based on modelling	Antenatal care	2.8 million pregnant women screening for syphylis, 7149 +ve
Ndibazza et al. [219]	2010	April 2003-November 2005	Uganda	Randomised placebo-controlled double-blinded	Antenatal care	2507 pregnant women
Ponmozhi et al. [220]	2017	December 2012-August 2014	India	Randomised double-blind placebo-controlled parallel arm superiority trial	Tertiary referral hospital	100 women below 16w GA with any risk factors for pre-eclampsia
Rempis et al. [95]	2017	February-December 2013	Uganda	Cross-sectional	District hospital	412 mother-newborn pairs
Salam et al. [76]	2014	May 2013	22 LMICs	Systematic literature review and meta-analysis	Community	32 studies
Unger et al. [75]	2015	November 2009-February 2013	Papua New Guinea	Parallel group randomised controlled trial	Antenatal care	2021 pregnant women <26w GA
Westen et al. [97]	2014	March 2008-February 2009	Tanzania	Randomised controlled non-inferiority trial	2 rural hospitals	176 women requiring Caesarean-section

**Table 5:** Maternal interventions- characteristics of individual studies (nutrition supplements)

Authors	Year of publication	Dates of study	Location	Study type	Study setting	Population
Bhutta et al. [148]	2013	N/A	LMICs	Review including evidence from the literature and de novo evidence	Community	Pregnant women
Muriel et al. [36]	2016	March 2010-June 2011	India	Case-control	Teaching hospital	Randomly selected women attending ANC- 100 intervention and 100 control
Haider and Bhutta [47]	2017	11-Mar 2015	LMICs	Cochrane review	Antenatal care	1 437 791 pregnant women in 17 studies
Haider et al. [51]	2013	31-May 2012	LMICs	Systematic literature review and meta-analysis	Antenatal care	12 932 pregnant women in 21 studies
He et al. [52]	2016	2010-2015	China	Retrospective cohort	Antenatal care	1 535 066 pregnant women
Hemminki et al. [53]	2016	June 2007- October 2008	Mozambique	Randomised control trial	2 health centres	Pregnant women: 2142 intervention and 2184 control
Hossain et al. [54]	2018	September 2010-May 2011	Pakistan	Randomised control trial	University teaching hospital	193 pregnant women
Janmohamad et al. [55]	2016	Unspecified	Cambodia	Cluster randomised controlled trial	Community (75 villages)	333 intervention and 214 control pregnant women
Kang et al. [56]	2017	2007 – 2012	Tibet	Prospective cohort	2 high altitude rural communities	1149 pregnant women
Kiondo et al. [57]	2014	November 2011-June2012	Uganda	Randomised placebo-controlled trial	Tertiary referral hospital	932 women age 15-42 GA 12-22w (466 intervention and 466 placebo)
Li et al. [37]	2014	October 1993- December 1996	China (2 provinces)	Prospective cohort	Antenatal care	207 936 singleton pregnancies from GA to 20w
Liu et al. [38]	2013	May 2006-April 2009	China	Randomised double-blinded controlled trial	5 counties	18 775 nulliparous pregnancy women without anaemia
McCauley et al. [39]	2016	30 March 2016	LMICs	Cochrane review	Antenatal care	Pregnant women in 19 trials
Mojibian et al. [40]	2015	2010-2012	Iran	Randomised control trial	2 prenatal clinics	500 pregnant women
Mosha et al. [41]	2016	August 2001-July 2004	Tanzania	Prospective cohort	Community	7634 pregnant women
Nossier et al. [42]	2015	February 2007-September 2009	Egypt	Double-blind placebo-controlled, parallel group randomised trial	Antenatal care	675 pregnant women
Ota et al. [43]	2015	31 October 2014	LMICs	Cochrane review	Antenatal care	21 RCTs including >17000 women
Pena-Rosas et al. [44]	2015	March 2012	LMICs	Systematic literature review and meta-analysis	Antenatal care	4072 women (from 18 trials)
Ramakrishnan et al. [45]	2016	November 2011-September 2013	Vietnam	Randomised double-blinded controlled trial	Antenatal care	1813 pregnant women
Sablok et al. [46]	2015	2010-2012	India	Randomised control trial	Tertiary referral hospital	180 pregnant women (60 control, 120 intervention)
Smith et al. [48]	2017	July 2015	14 LMICs	Systematic literature review and meta-analysis	Antenatal care	112 953 women
West et al. [49]	2014	4 December 2007- 30 August 2012	Bangladesh	Cluster randomised double masked control trial	Antenatal care	44 567 pregnancies
Zheng et al. [50]	2016	1999-2012	China	Prospective cohort	Community	231 179 deliveries seen in 1st trimester (excluding singletons weighing <1000g or >5000g)

**Table 6:** Maternal interventions- characteristics of individual studies (community groups)

Authors	Year of publication	Dates of study	Location	Study type	Study setting	Population
Colbourn et al. [58]	2015	1 October 2008- 31 December 2010	Malawi	Prospective cohort	Community	729 community groups
Fottrell et al. [59]	2016	1 January 2009- 30 June 2011	Bangladesh	Cluster randomised controlled trial	Community	19 301 pregnant women
Lassi and Bhutta [29]	2015	May 2014	India, Bangladesh, Pakistan, Nepal, China, Zambia, Malawi, Tanzania, South Africa, Ghana	Cochrane review	Community	26 studies
Lassi et al. [25]	2016	January 2015	LMICs	Systematic literature review and meta-analysis	Community	N/A
Lewycka et al. [63]	2013	2005-2009	Malawi	Cluster randomised controlled trial	Community	185 888 women with 26 262 births
Mbuagbaw et al. [26]	2016	June 2015	LMICs	Cochrane review	Community	34 trials (NB 5 of these in HICs)
Pasha et al. [61]	2013	March 2009-30 September 2011	Pakistan, Kenya, Zambia, Guatemala and Argentina	Cluster randomised controlled trial	Community	55 712 intervention and 54 822 control over 106 clusters
Prost et al. [65]	2013	N/A	Bangladesh, Malawi, India and Nepal	Systematic literature review and meta-analysis	Community	119 428 births over 7 trials
Soubeiga et al. [66]	2014	December 2013	India, nepal, Bangladesh, Ghana, Malawi, Pakistan, Brazil, Argentina	Systematic literature review and meta-analysis	Community	307 018 pregnant women across 14 studies

**Table 7:** Neonatal interventions- characteristics of individual studies grouped by intervention type (infection prevention and treatment)

Authors	Year of publication	Dates of study	Location	Study type	Study setting	Population
Afjeh et al. [133]	2016	July 2011-June 2012	Iran	Prospective cohort	Tertiary referral perinatal centre	All VLBW newborns admitted to NICU that survived >2W (N = 145, 104/145 inborn)
Banupriya et al. [112]	2017	May 2013-November 2015	India	RCT	Tertiary referral centre	Neonates aged <28 d, GA>31 weeks, on significant enteral feeds, biochemical or microbioogical evidence of infection (N = 134). Excluded if already on Abx for sepsis.
Cleminson et al. [143]	2016	August 2015.	India, Egypt, Bangladesh, Turkey, Iran, Pakistan, Brazil	Systematic literature review & meta-analysis	Health facilities and community	1184 infants. 11 trials (9 from LMICs).
Debes et al. [109]	2013	December 2011.	Ghana, Nepal, India	Systematic literature review & meta-analysis	Health facilities and community	18 studies. 3 studies included in mortality analyses.
Dilli et al. [114]	2015	June 2011-June 2014	Turkey	RCT	5 tertiary referral centres	VLBW infants with GA<32 weeks and birthweight <1500g (N = 400). Neonates who died in 1st week of life were excluded.
Erdermir et al. [134]	2015	September 2010-September 2012	Turkey	RCT	Tertiary referral centre	Preterm newborns <35 weeks gestation and <24 old at time of admission (N = 197)
Fernandez-Carrocera et al [116]	2013	January 2007-June 2010	Mexico	RCT	Tertiary referral centre	Preterm newborns <1500g BW who were admitted to neonatal care
Guney-Varal et al. [113]	2017	“one year period”-dates not reported	Turkey	RCT	Tertiary referral centre	Infants <33 weeks GA and <1500g (N = 110)
Kaur et al. [117]	2015	May 2012-July 2013	India	RCT	Tertiary referral centre	Inborn neonates <2000g admitted to NICU in first 12h of birth with no maternal risk factors for sepsis (N = 130). Neonates who developed culture-proven sepsis within 72h of life were excluded.
Hosseini et al. [144]	2017	January 2013-June 2015	Iran	RCT	Tertiary referral centre	Preterm infants with birthweight <1500g admitted to NICU with suspected sepsis (N = 209)
Khan et al. [111]	2015	Not reported	Bangladesh, Philippines, India, Ghana, Mexico, Nepal, Brazil	Systematic literature review & meta-analysis	Health facilities and community	10 studies, 6 in LMICs.
Li et al. [135]	2015	January 2008-December 2013 (Retrospective: Jan 2008-Dec 2010, Prospective Jan 2011-Dec 2013)	China	Ambispective cohort	Tertiary referral centre	All newborns admitted to neonatology department with TTN and GA between 34 and 42 weeks (N = 1485).Excluded if BW<2000g.
Massawe et al. [130]	2018	Pre: Sept 2014-May 2015, Post: June 2015-June 2017	Tanzania	Pre/post implementation	Multi-site: 3 tertiary referral hospitals, 1 district hospital	Inpatient pregnant women and inborn preterm neonates. N = 3496 preterm babies (543 pre-implementation, 2953 post-implementation).
Nandhini et al. [122]	2016	Not reported	India	RCT	Tertiary referral centre	Enterally fed preterm neonates GA 28 - 34 weeks and BW>1000g.
Oncel et al. [115]	2014	February 2012-February 2013	Turkey	RCT	Tertiary referral centre	Preterm infants GA<33 weeks and birthweight <1501g who survived to feed enterally (N = 400)
Panigrahi et al. [103]	2017	Not reported	India	RCT	149 randomly chosen villages in 1 state	All births identified in study villages (N = 4556). Excluded neonates <35 weeks gestation or <2000g birthweight.
Patel et al. [104]	2018	Pre: December 2013-October 2014. Post: November 2014-December 2015.	India	Retrospective pre/post implementation	Tertiary referral centre	Preterm infants <35 weeks GA admitted to neonatal unit (N = 199, Pre:145, Post: 44)
Pinto et al. [145]	2013	Pre: January 2006-December 2007. Post: January 2008-December 2008.	Brazil	Pre/post implementation	Tertiary referral centre	Newborns <1500g admitted to NICU and commenced on broad-spectrum Abx for suspected sepsis (N = 136, Pre: 91, Post: 45).
Salam et al. [146]	2015	July 2011-January 2012	Pakistan	RCT	Tertiary referral centre	All infants <37 weeks GA with birthweight >749g. Excluded if severe RDS, skin infection or life-threatening congenital abnormality (N = 258)
Salam et al. [136]	2013	December 2012.	India, Egypt, Bangladesh, Pakistan, Brazil	Systematic literature review & meta-analysis	Health facilities and community	7 studies. 689 infants from 3 studies included in meta-analysis.
Sankar et al. [137]	2013	Not reported	Malawi, Egypt, Nepal, Pakistan, Brazil, South Africa, India, Zimbabwe	Systematic literature review & meta-analysis	Health facilities and community	9 studies. 5 studies provided data on all-cause mortality.
Santana et al. [105]	2017	August 2014-October 2015	Brazil	Retrospective cohort	Tertiary referral centre	All consecutive neoantes GA<37 weeks born at study site and admitted for at least 5 d to NICU (N = 300). Neonates with “trans-placental infection potential” excluded.
Sazawal et al. [138]	2016	May 2011-August 2014	Zanzibar	RCT	Population level	All newborn babies born on island aged 1h - 48h without congenital malformations (N = 36911)
Schmidt et al. [139]	2018	Pre: 2010-2012. Post: October 2013-July 2016	Lao	Retrospective pre/post implementation	5 provincial hospitals with highest mortality rate	Newborns admitted to pediatric ward or NICU in participating sites. (N = 3889, Pre N = 1673 Post N = 2216).
Semrau et al. [147]	2016	Feb 2011-Jan 2013	Zambia	RCT (cluster)	90 community heath facility-based clusters (must provide routine antenatal services and at least 160 annual births in catchment area. 12 urban, 78 rural))	Pregnant women (Aged >14, in second or third trimester) attending antenatal clinics or identified during community outreach activities (N = 42 356)
Serce et al. [106]	2013	October 2012-November 2011.	Turkey	RCT	Tertiary referral centre	Preterm newborns admitted to NICU (GA<33 weeks, birthweight <1501g). Excluded if death occurred in first 24h (N = 208)
Shabaan et al. [140]	2017	August 2013-June 2015	Egypt	RCT	Tertiary referral centre	Inborn and outborn neonates with late-onset sepsis caused by gram negative bacteria sensitive to meropenem. Excluded SGA neonates and those with congenital infection (N = 102).
Soofi et al. [141]	2017	April 2009-December 2012	Pakistan	RCT (cluster)	Rural district with population approx. 0.56 million. Clustered into 27 clusters served by an individual functional primary care facility.	All households in study area
Van Niekerk et al. [123]	2015	July 2011-August 2012	South Africa	RCT	Tertiary referral centre	HIV-exposed and HIV-unexposed infants <34 weeks GA anc <1250g who were breast-milk fed delivered in study site
Zhou et al. [142]	2013	Pre: February 2006-January 2007. Partial intervention: August 2008-July 2009. Full intervention: January 2010-December 2010	China	Pre/post implementation	Tertiary referral centre	All neonates who received mechanical ventilation for at least 48 h and were hospitalized in NICU for at least 5 d (N = 491, Pre: 106, Partial: 169, Full: 216)

**Table 8:** Neonatal interventions- characteristics of individual studies grouped by intervention type (respiratory support)

Authors	Year of publication	Dates of study	Location	Study type	Study setting	Population
Ali et al. [150]	2016	December 2015.	LMICs (Iran, Turkey, China)	Systematic literature review & meta-analysis	Health facilities	400 infants. 4 studies
Boo et al. [151]	2016	January-December 2013	Malaysia	Retrospective cohort	36 neonatal intensive care units in the Malaysian National Neonatal Registry	All VLBW neonates born in participating hospitals and admitted to NICU (N = 2823)
Ceylan et al. [162]	2014	2009.- 2011	Turkey	Prospective case/control	Tertiary referral centre	Infants with RDS with GA<33 weeks (N = 109)
Crivceanscala et al. [167]	2017	2013-2016	Moldova	Prospective cohort	Tertiary referral centre	Neonates <34 weeks GA with RDS
Daga et al. [168]	2014	June-October 2012	India	Pre/post implementation	Tertiary referral centre	Neonates admitted to NICU with RDS during tiem period (N = 140, Pre = 56, Post = 84)
Dilmen et al. [169]	2014	June 2009-June 2010	Turkey	RCT	6 tertiary referral centres	All infants between 25 and 30 weeks GA who were not intubated in the delivery room (N = 159)
Goncalves-Ferri et al. [170]	2014	June 2008-December 2009	Brazil	RCT	5 tertiary referral centres	All neonates with birthweight 1000-1500g and no major congenital malformations who were not intubated or extubated <15 min after birth (N = 197)
Guinsburg et al. [164]	2018	2014-2015	Brazil	Prospective cohort	20 tertiary referral centres part of Brazilian Network on Neonatal Research	Inborn infants ventilated at birth GA 23-33 weeks & birthweight 400-1499g (N = 1962)
Kanmaz et al. [165]	2013	December 2010-December 2011	Turkey	RCT	Tertiary referral centre	Inborn infants GA<32 weeks with RDS (N = 200). Excluded if required intubation in delivery room.
Kawaza et al. [171]	2016	January-October 2012	Malawi	Non-randomized convenience sample study	Tertiary referral hospital	Neonates weighing >1000g and presenting with respiratory distress syndrome (N = 87 (62 bCPAP, 25 controls)
Kong et al. [172]	2016	2012-2013	China	Prospective cohort	9 NICUs part of Neonatal Research Network	Infants GA 25 weeks to <28 weeks or infants GA 28 weeks to <32 weeks with at least 3 of 1) maternal diabetes 2) male infants 3) multiple births 4)no/insufficient ACS 5)emergency intubation requirement after birth 6) severe RDS. (N = 254)
Kumar et al. [173]	2017	June 2014-June 2016	India	RCT	Tertiary referral centre	Preterm neonates GA<31 weeks who were intubated immediately postnatally for RDS and on mechanical ventilation for minimum 24 h (N = 156)
Martin et al. [152]	2014	March 2014.	Malawi, South Africa	Systematic literature review & meta-analysis	Health facilities	582 infants from 3 studies used mortality analysis.
Mazmanyan et al. [153]	2016	Not reported	Armenia	RCT	Tertiary referral centre	All infants born <37 weeks of age (N = 125)
Myhre et al. [154]	2016	Pre: November 2007-April 2009. Post: November 2009-April 2011.	Kenya	Retrospective pre/post intervention	Rural district hospital	All neonates GA<37 weeks with RDS (N = 118, Pre = 46, Post = 72)
Nahimana et al. [155]	2015	Febrary 2013-October 2013	Rwanda	Retrospective cohort	Multi-site: 3 rural district hospitals	All preterm/very low birthweight infants admitted to neonatology units at study sites (N = 136 of 862 admissions)
Niknafs et al. [156]	2014	June 2012-December 2012	Iran	Prospective cohort	Multi-site: 2 tertiary referral NICUs	Inborn infants suffering from RDS in either study site
Ntigurirwaet al. [157]	2017	Feb 2012-Jan 2014	Rwanda	Pre/post implementation	Multi-site: 2 university hospitals, 2 district hospitals	Neonates admitted to neonatal units at study sites
Rebello et al. [158]	2014	August 2005-August 2007	Brazil	RCT	Multi-site - 19 neonatal intensive care units	Preterm neonates born in study centres with birthweight 501-1500g, age <25 h of life, undergoing mechanical ventilation and with clinical & radiological diagnosis of RDS. (N = 327)
Rezzonico et al. [167]	2015	Pre: May 2006 0 December 2006. Post: May 2008-December 2008.	Nicaragua	Pre/post implementation	Tertiary referral centre	Newborns admitted to NICU “with a history of ventilatory assistance” ie, requiring ventilation resuscitation at birth. (N = 613, Pre: 230, Post: 383.
Sankar et al. [159]	2016	June 2013.	Mexico, Turkey, South Africa, Malaysia, Brazil, Chile, Argentina, Malaysia, India, China, Peru, Uruguay, Iran	Systematic literature review & meta-analysis	Health facilities and community	38 studies. 2 RCTs and 22 observational studies reporting on mortality.
Say et al. [160]	2016	May 2014-November 2014	Turkey	RCT	Tertiary referral centre	Preterm infants GA 26-32 weeks with diagnosed RDS (N = 149)
Schmidt et al. [139]	2018	Pre: 2010-2012. Post: October 2013-July 2016	Lao	Retrospective pre/post implementation	5 provincial hospitals with highest mortality rate	Newborns admitted to pediatric ward or NICU in participating sites. (N = 3889, Pre N = 1673 Post N = 2216).
Thukral et al. [161]	2016	December 2014	Fiji, South Africa, Malawi, India, Colombia, Nepal, Malaysia, India	Systematic literature review & meta-analysis	Secondary referral centres	22 studies
Zubizaretta et al. [163]	2016	2005-2011	Argentina, Brazil, Chile, Paraguay, Peru, Uruguay	Prospective cohort	25 NICUs	All infants with birthweight 500-1500g admitted to one of study NICUs. (N = 634 matched pairs ie, total 1268).

**Table 9:** Neonatal interventions- characteristics of individual studies grouped by intervention type (cardiovascular support)

Authors	Year of publication	Dates of study	Location	Study type	Study setting	Population
Ohlsson et al. [183]	2018	November 2017	Jordan, Iran, China, India, Egypt, Turkey	Systematic literature review & meta-analysis	Health facilities	9 studies (8 from LMICs). 272 infants from 3 studies all from LMICs included in mortality meta-analysis.
Sadeck et al. [182]	2014	January 2010-December 2011	Brazil	Retrospective cohort	16 tertiary referral centres part of Brazilian Network on Neonatal Research	Neonates GA<33 weeks, birthweight 400-999g, echo diagnosis of PDA. Neonates who died in first 3 d of life were excluded. (N = 494)

**Table 10:** Neonatal interventions- characteristics of individual studies grouped by intervention type (Health systems, training and guidelines)

Authors	Year of publication	Dates of study	Location	Study type	Study setting	Population
Ashish et al. [186]	2016	July 2012-September 2013	Nepal	Pre/post implementation	Tertiary referral centre	All women delivering at >21 weeks gestation in the study site (N = 25 108 deliveries, Pre = 9588, Post = 15 520).
Bellad et al. [194]	2016	Pre: November 2011-October 2012 Post: November 2012-October 2013	India and Kenya	Pre/post implementation	Multi-site birth cohorts. Intervention delivered at facilities that provided 24/7 delivery services. Belgaum: 19 primary facilities, 12 secondary facilities and 2 tertiary. Nagpur: 2 primary, 4 secondary, 9 tertiary. Kenya: 18 primary and 5 secondary.	All babies born >1500g in all birth cohort study sites. N = 70 704 (Pre: 35 595, Post: 35 109)
Boone et al. [177]	2017	August 2008-November 2011	India	RCT (cluster)	Villages with populations <2500	Villages = clusters. (N = 464). Randomised 1:1. Eligible women: <50 y old, married, not sterilised (N = 29 669, 15 532 intervention, 14 137 control)
Cavicchiolo et al. [197]	2016	January 2013-December 2014	Mozambique	Retrospective pre/post implementation	Tertiary referral centre	All newborns admitted to neonatal unit (N = 4276 newborns, Pre = 2118, Post = 2158)
Colbourn et al. [178]	2013	October 2008-December 2010	Malawi	RCT (cluster)	Clusters of approx 4000 people. All health facilities in districts included apart from those providing CEmOC, those not providing BEmOC (ie, dispensaries) and non-functional facilities.	All pregnant women in study areas.
Dempsey et al. [184]	2015	March 2015.	Argentina, DRC, Guatemala, India, Pakistan, Zambia, Kenya, China	Systematic literature review & meta-analysis	Health facilities	66 162 neonates from 3 studies
Fottrell et al. [59]	2013	January 2009-June 2011	Bangladesh	RCT (cluster)	18 unions in 3 districts	All women residing in clusters. Accounting for N = 19301 births during final 24 months of intervention.
Gilbert et al [193]	2014	Pre: July 2008-June 2009. Post: October 2009-September 2010.	Brazil	Prospective Pre/post implementation	5 tertiary referral centres	Infants with BW<1501g or GA<35 weeks admitted to study NICUs (N = 1242, Pre: 679, Post: 563).
Gomez et al. [86]	2018	March 2014-February 2017. Each facility enrolled for 18 mo. Data collected 6 mo pre-intervention and 12 mo post-intervention.	Ghana	Cluster randomized trial (sites randomised to 1 of 4 implementation waves)	40 public & mission hospitals - 3 regional hospitals, 38 district hospitals and 1 polyclinic	All newborns delivered at particpating facilities (N = 105 850. Pre: 38 192, Post months 1-6: 36 160, Post months 7-12: 31 498)
Goudar et al. [185]	2013	Pre: October 2009-March 2010, Post: March 2010-Septmeber 2010	India	Pre/post implementation	Public health clinics, district hospitals, urban hospitals. (98% in district hospital and KLES hospital in Belgaum)	All babies delivered at study sites (N: pre = 4187, post = 5411)
Harris et al. [198]	2018	Unclear on dates. NMR audited 3 mo pre-intervention and then 3 mo post implementation and for 3 mo at the same time of year 3 y and 6 y post implementation.	Uganda	Pre/post implementation	District mission hospital	All newborns admitted to neonatal unit
Massawe et al. [130]	2018	Pre: Sept 2014-May 2015, Post: June 2015-June 2017	Tanzania	Pre/post implementation	Multi-site: 3 tertiary referral hospitals, 1 district hospital	Inpatient pregnant women and inborn preterm neonates. N = 3496 preterm babies (543 pre-implementation, 2953 post-implementation).
Hosseini et al. [144]	2017	January 2013-June 2015	Iran	RCT	Tertiary referral centre	Preterm infants with birthweight <1500g admitted to NICU with suspected sepsis (N = 209)
Kakkad et al. [199]	2014	Pre: Sept 2011-Aug 2012. Post: Sept 2012-Aug 2013.	India	Retrospective pre/post intervention	Tertiary referral centre	All neonates admitted to NICU (N = 7568, Pre = 3455, Post = 4133)
Kirkwood et al. [181]	2013	November 2008-December 2009	Ghana	RCT (cluster)	7 rural districts split into 98 clusters based on community-based surveillance volunteer (CBSV) supervisory zones	All pregnancies that ended in a livebirth or stillbirth in study areas (N = 18609 eligible pregnancies).
Leng et al. [128]	2016	Pre: January 2010-March 2011. Post: October 2012-September 2013.	China	Ambispective cohort	Tertiary referral centre	Outborn VLBW neonates referred to study NICU within 12h of birth (pre: 86, post: 86)
Lewcyka et al. [63]	2013	December 2004-December 2010	Malawi	RCT (cluster)	48 community clusters	48 equal-sized clusters of approx. 3000 people randomly allocated to 1 of 4 groups. All women aged 10-49 included: (total N = 55 931). Outcomes monitored for N = 26 262 births.
Li et al. [135]	2015	January 2008-December 2013 (Retrospective: Jan 2008-Dec 2010, Prospective Jan 2011-Dec 2013)	China	Ambispective cohort	Tertiary referral centre	All newborns admitted to neonatology department with TTN and GA between 34 and 42 weeks (N = 1485).Excluded if BW<2000g.
Massawe et al. [130]	2018	Pre: Sept 2014-May 2015, Post: June 2015-June 2017	Tanzania	Pre/post implementation	Multi-site: 3 tertiary referral hospitals, 1 district hospital	Inpatient pregnant women and inborn preterm neonates. N = 3496 preterm babies (543 pre-implementation, 2953 post-implementation).
Mduma et al. [187]	2015	Pre: February 2010-January 2011. Post: February 2011-January 2012	Tanzania	Pre/post implementation	Rural referral hospital	All deliveries at study site (N = 9708, Pre - N = 4894, Post- N = 4814)
Msemo et al. [196]	2013	Pre: July 2009-August 2009, Post: Sept 2009-March 2012	Tanzania	Pre/post implementation	Multi-site: 3 tertiary referral hospitals, 4 associated regional hospitals, 1 district hospital	Newborns with a 5-min Apgar score <7 and requirement for facemask ventilation with BW>750g or SB>1000g (N = 85 338, 7969 pre, 77 369 post)
Namazzi et al. [188]	2015	2007-2011 (pre: 2007-2009, post: 2009-2011)	Uganda	Pre/post implementation	Demographic & health surveillance Site. 20 health facilities targeted: 1 district hospital, 1 level IV health centre, 6 level III health centres and 12 level II health centres	Health facilities within district serving a population of around 70 000
Ntigurirwa et al. [157]	2017	Feb 2012-Jan 2014	Rwanda	Pre/post implementation	Multi-site: 2 university hospitals, 2 district hospitals	Neonates admitted to neonatal units at study sites
Opiyo et al. [189]	2015	February 2015.	Kenya, Sri Lanka	Systematic literature review & meta-analysis	Primary health facilities	2 studies
Pammi et al. [195]	2016	April 2015.	Argentina, DRC, Guatemala, India, Pakistan, Zambia, China, Kenya	Systematic literature review & meta-analysis	Health facilities and community	14 studies. 28 923 infants from 3 studies included in meta-analysis.
Patel et al. [104]	2018	Pre: December 2013-October 2014. Post: November 2014-December 2015.	India	Retrospective pre/post implementation	Tertiary referral centre	Preterm infants <35 weeks GA admitted to neonatal unit (N = 199, Pre:145, Post: 44)
Pinto et al. [145]	2013	Pre: January 2006-December 2007. Post: January 2008-December 2008.	Brazil	Pre/post implementation	Tertiary referral centre	Newborns <1500g admitted to NICU and commenced on broad-spectrum Abx for suspected sepsis (N = 136, Pre: 91, Post: 45).
Schmidt et al. [139]	2018	Pre: 2010-2012. Post: October 2013-July 2016	Lao	Retrospective pre/post implementation	5 provincial hospitals with highest mortality rate	Newborns admitted to pediatric ward or NICU in participating sites. (N = 3889, Pre N = 1673 Post N = 2216).
Singh et al. [180]	2013	April 2008-December 2009	Ghana	Interrupted time-series	Facilities in 4 rural districts - 25 health centres and 2 district hospitals	Women and children receiving care at these facilities (inclusion/exclusion unclear)
Soofi et al. [141]	2017	April 2009-December 2012	Pakistan	RCT (cluster)	Rural district with population approx. 0.56 million. Clustered into 27 clusters served by an individual functional primary care facility.	All households in study area
Sousa et al. [190]	2015	Not reported	Guatemala, DRC, Pakistan, Zambia, India, Uganda, Kenya, Pakistan, Tanzania, Malawi, Bangladesh	Systematic literature review & meta-analysis	Health facilities and community	14 studies.
Turner et al. [191]	2013	January 2008-December 2011	Thai/Myanmar border	Retrospective cohort	NICU in refugee camp	All newborns admitted to special care baby unit GA>27 weeks whose mother had ANC In refugee camp (N = 923).
Wrammert et al. [192]	2017	July 2012-September 2013	Nepal	Prospective pre/post implementation	Tertiary referral centre	All women delivering at >21 weeks gestation in the study site (N = 24 665 deliveries, Pre = 9390, Post = 15 275).
Zhou et al. [142]	2013	Pre: February 2006-January 2007. Partial intervention: August 2008-July 2009. Full intervention: January 2010-December 2010	China	Pre/post implementation	Tertiary referral centre	All neonates who received mechanical ventilation for at least 48 h and were hospitalized in NICU for at least 5 d (N = 491, Pre: 106, Partial: 169, Full: 216)
Zonneveld et al. [200]	2017	Pre: July 2014-March 2015. Post: March 2015-December 2015	Suriname	Retrospective pre/post implementation.	Tertiary referral centre	All inborn & outborn neonates admitted to study site (N = 601, Pre: 320, Post:281)

**Table 11:** Neonatal interventions- characteristics of individual studies grouped by intervention type (feeding and nutrition)

Authors	Year of publication	Dates of study	Location	Study type	Study setting	Population
Banupriya et al. [112]	2017	May 2013-November 2015	India	RCT	Tertiary referral centre	Neonates aged <28 d, GA>31 weeks, on significant enteral feeds, biochemical or microbioogical evidence of infection (N = 134). Excluded if already on Abx for sepsis.
Debes et al. [109]	2013	December 2011	Ghana, Nepal, India	Systematic literature review & meta-analysis	Health facilities and community	18 studies. 3 studies included in mortality analyses.
Deshpande et al. [102]	2017	January 2017	Egypt, Brazil, Iran, Turkey, China, Mexico, India, Colombia, Thailand, South Africa	Systematic literature review & meta-analysis	Health facilities and community	4783 infants. 23 studies.
Dilli et al. [114]	2015	June 2011-June 2014	Turkey	RCT	5 tertiary referral centres	VLBW infants with GA<32 weeks and birthweight <1500g (N = 400). Neonates who died in 1st week of life were excluded.
Edmond et al. [101]	2015	August 2010-November 2011	Ghana	RCT	7 rural districts	Newborns identified at home or facilities on day of birth or in the next 2 d, more than 2h old and able to feed orally. (N = 22955)
English et al. [110]	2017	January 2014	LMICs	Review of systematic reviews	Health facilities and community	3 systematic reviews of 2 neonatal interventions
Fernandez-Carrocera et al [116]	2013	January 2007-June 2010	Mexico	RCT	Tertiary referral centre	Preterm newborns <1500g BW who were admitted to neonatal care
Guney-Varal et al. [113]	2017	“one year period”-dates not reported	Turkey	RCT	Tertiary referral centre	Infants <33 weeks GA and <1500g (N = 110)
Gurpreet et al. [117]	2015	May 2012-July 2013	India	RCT	Tertiary referral centre	Inborn neonates <2000g admitted to NICU in first 12h of birth with no maternal risk factors for sepsis (N = 130). Neonates who developed culture-proven sepsis within 72h of life were excluded.
Khan et al. [111]	2015	Not reported	Bangladesh, Philippines, India, Ghana, Mexico, Nepal, Brazil	Systematic literature review & meta-analysis	Health facilities and community	10 studies, 6 in LMICs.
Lund et al. [118]	2014	February 2008-November 2008 (stopped early)	Guinea-Bissau	RCT	All districts of capital city	All newborn males weighing <2.5kg at discharge from maternity ward of the national hospital (N = 232 at point trial stopped)
Mahallei et al. [119]	2016	Not reported	Iran	RCT	Tertiary referral centre	Preterm neonates GA<32 weeks & birthweight <1500g (N = 120)
Masanja et al. [120]	2015	August 2010-March 2013	Tanzania	RCT	Community	Dar Es Salaam - mothers/newborns from 1 antenatal clinics/labour wards. Morogoro region - nested within health and demographic surveillance system. Newborns eligible if able to feed orally (same criteria as other Neovit studies) (N = 31 999)
Mazumder et al. [121]	2015	June 2010-July 2012	India	RCT	2 districts (approx. 2.1 million people)	Pregnant women identified through 3 moly surveillance programme. All neonates who were able to feed orally were eligible (N = 44, 984).
Nandhini et al. [122]	2016	Not reported	India	RCT	Tertiary referral centre	Enterally fed preterm neonates GA 28 - 34 weeks and BW>1000g.
Oncel et al. [115]	2014	February 2012-February 2013	Turkey	RCT	Tertiary referral centre	Preterm infants GA<33 weeks and birthweight <1501g who survived to feed enterally (N = 400)
Panigrahi et al. [103]	2017	Not reported	India	RCT	149 randomly chosen villages in 1 state	All births identified in study villages (N = 4556). Excluded neonates <35 weeks gestation or <2000g birthweight.
Patel et al. [104]	2018	Pre: December 2013-October 2014. Post: November 2014-December 2015	India	Retrospective pre/post implementation	Tertiary referral centre	Preterm infants <35 weeks GA admitted to neonatal unit (N = 199, Pre:145, Post: 44)
Santana et al. [105]	2017	August 2014-October 2015	Brazil	Retrospective cohort	Tertiary referral centre	All consecutive neoantes GA<37 weeks born at study site and admitted for at least 5 d to NICU (N = 300). Neonates with “trans-placental infection potential” excluded.
Serce et al. [106]	2013	October 2012-November 2011	Turkey	RCT	Tertiary referral centre	Preterm newborns admitted to NICU (GA<33 weeks, birthweight <1501g). Excluded if death occurred in first 24h (N = 208)
Tali et al. [108]	2016	Not reported	India	RCT	Tertiary referral centre	Neonates admitted to NICU weighing 501-1500g. Excluded if GI or other severe congenital malformations, or if severely unwell such that not able to feed (severe sepsis, shock etc) (N = 120).
Van Niekerk et al. [123]	2015	July 2011-August 2012	South Africa	RCT	Tertiary referral centre	HIV-exposed and HIV-unexposed infants <34 weeks GA anc <1250g who were breast-milk fed delivered in study site

**Table 12:** Neonatal interventions- characteristics of individual studies grouped by intervention type (community mobilisation)

Authors	Year of publication	Dates of study	Location	Study type	Study setting	Population
Boone et al. [177]	2017	August 2008-November 2011	India	RCT (cluster)	Villages with populations <2500	Villages = clusters. (N = 464). Randomised 1:1. Eligible women: <50 y old, married, not sterilised (N = 29 669, 15 532 intervention, 14 137 control)
Colbourn et al. [178]	2013	October 2008-December 2010	Malawi	RCT (cluster)	Clusters of approx 4000 people. All health facilities in districts included apart from those providing CEmOC, those not providing BEmOC (ie, dispensaries) and non-functional facilities.	All pregnant women in study areas.
Fottrell et al. [59]	2013	January 2009-June 2011	Bangladesh	RCT (cluster)	18 unions in 3 districts	All women residing in clusters. Accounting for N = 19 301 births during final 24 mo of intervention.
Kirkwood et al. [181]	2013	November 2008-December 2009	Ghana	RCT (cluster)	7 rural districts split into 98 clusters based on community-based surveillance volunteer (CBSV) supervisory zones	All pregnancies that ended in a livebirth or stillbirth in study areas (N = 18 609 eligible pregnancies).
Lassi et al. [64]	2015	May 2014	LMICs	Systematic literature review & meta-analysis	Community	26 studies. 302 646 infants from 21 studies used in NMR meta-analysis
Lassi et al. [176]	2016	January 2015	LMICs	Systematic literature review & meta-analysis	Community	310 652 participants. 58 studies. 20 studies (N = 248 848) included in mortality analyses.
Lewcyka et al. [63]	2013	December 2004-December 2010	Malawi	RCT (cluster)	48 community clusters	48 equal-sized clusters of approx. 3000 people randomly allocated to 1 of 4 groups. All women aged 10-49 included: (total N = 55 931). Outcomes monitored for N = 26 262 births.
Persson et al. [179]	2013	July 2008-June 2011	Vietnam	RCT (cluster)	90 of 187 communes in 1 province	All women and children in participating communes. N = 22 561 births registered during study period.
Sarbani Roy et al. [131]	2013	November 2004-July 2011	India	Prospective cohort	India: 2 states, community DHS data	All births, stillbirths and neonatal deaths in 36 geographical clusters recorded via surveillance system
Singh et al. [180]	2013	April 2008-December 2009	Ghana	Interrupted time-series	Facilities in 4 rural districts - 25 health centres and 2 district hospitals	Women and children receiving care at these facilities (inclusion/exclusion unclear)
Soofi et al. [141]	2017	April 2009-December 2012	Pakistan	RCT (cluster)	Rural district with population approx. 0.56 million. Clustered into 27 clusters served by an individual functional primary care facility.	All households in study area

## RESULTS

A total of 81 studies of maternal interventions (68 implementation studies and 13 systematic reviews) were included in analysis. Fifty-five studies reported on data from Sub-Saharan Africa, 25 from South Asia, 20 from East Asia and Pacific, 15 from the Middle East and North Africa, 12 from Latin America and the Caribbean and 2 from Europe and Central Asia.

Ninety-eight studies of neonatal interventions (79 implementation studies, 19 systematic reviews) were included in analyses. 36 reported data from Sub-Saharan Africa, 35 from South Asia, 12 from East Asia and Pacific, 10 from the Middle East and North Africa, 14 from Latin America and the Caribbean, and 12 from Europe and Central Asia.

### Maternal interventions

**Tables 2-7** show the study characteristics and details of the maternal interventions.

### Antenatal care

Thirteen studies were identified reporting on the impact of antenatal care [17–28]. Five of these showed antenatal care was associated with a reduction in stillbirth [17,20,22,25,26], two of which also showed reduction in neonatal mortality [25,26], two studies found antenatal care reduced a composite outcome of stillbirth, preterm birth and low birthweight [18,19], and four studies showed no significant difference in our outcomes [21,23,24,27]. A meta-analysis found antenatal care reduced both stillbirth (relative risk (RR) = 0.82, 95% Confidence Interval (CI) = 0.73-0.93) and neonatal mortality (RR = 0.80, 95% CI = 0.72-0.8) [29].

Two studies evaluated the effect of introducing routine ultrasound scanning during antenatal care [27,30]. Neither found any improvement in stillbirth or preterm birth reduction, although both led to increased identification of fetal problems. This included a large cluster randomised controlled trial encompassing 51 423 births, with stillbirth reduction (RR = 1.09, 95% CI = 0.97-1.23) and neonatal mortality reduction (RR = 0.99, 95% CI = 0.86-1.14) [30].

### Genito-urinary infection management

Three randomised-controlled trials reported on genito-urinary infection management, focussing on testing and treating for bacterial vaginosis during the second trimester [31-33]. Two of these trials found no significant difference in any outcomes[31,32] however one trial comparing bacterial vaginosis treatment vs placebo in women with abnormal vaginal flora found a significant reduction in preterm birth (RR = 1.65, 95% CI = 1.04-2.63) [33]. A prospective cohort study in China evaluating the introduction of routine syphilis testing at first antenatal appointment, estimated a 39.4% reduction in stillbirth and an 8.8% reduction in preterm birth [34].

### Nutritional supplements

Twenty three studies reported on antenatal nutritional supplements [35-57]. Folic acid, zinc, calcium and multiple-micronutrient supplementation were all found to reduce preterm birth, whereas vitamin D, vitamin C and iron had no effect when given alone.

Three studies reported folic acid supplementation before conception and during pregnancy [37,50,52]. All found a significant reduction in preterm birth and this effect size was increased if taken from 3 months before the last menstrual period [52]. There were two studies with zinc supplementation, a Cochrane review on zinc alone showed a significant reduction in preterm birth rate (RR = 0.86, 95% CI = 0.76-0.97) [43] and a RCT using zinc in a combination tablet with other multivitamins was associated with a reduction in stillbirth, preterm birth and early neonatal mortality [42]. There were two RCTs with vitamin D supplementation one of which found no significant effect[40] and the other found a reduction in preterm birth [46]. Vitamin C alone was found to have no difference in outcomes [57] however another study combining vitamin E and C observed preterm birth reduction [36]. Three studies used iron supplement regimes, pre-natal iron had no effect [51], neither did testing and treating anaemia [53]. Although low dietary iron was significantly associated with stillbirth and preterm birth (RR = 0.12, 95% CI = 0.036-0.377) and early neonatal mortality (RR = 0.23 95% CI = 0.15-0.35) [41], a systematic literature review comparing daily with intermittent iron found no significant difference in preterm birth (odds ratio (OR)  = 1.82, 95% CI = 0.75-4.4) [44]. Low dietary calcium was associated with increased preterm birth (RR = 0.76, 95% CI = 0.65-0.88) [41] and accordingly calcium supplementation was associated with a reduction in preterm birth (RR = 0.76, 95% CI = 0.60-0.97) [35].

Seven papers compared maternal micronutrient supplementation (MMN) with combined iron and folic acid supplementation [35,38,45,47-49,56]. All of these studies, including a Cochrane review, found significant reduction in both preterm birth and stillbirth with MMN compared with iron and folic acid alone. This effect was even more pronounced in women with anaemia or low body mass index (BMI) [47,48].

### Community groups

We identified nine papers including two systematic literature reviews and two Cochrane reviews evaluating the impact of community groups, all of which demonstrated significant reductions on stillbirth and neonatal mortality [26,29,58-63]. Three of these used community based intervention packages to deliver antenatal care- for example upscaling home visits by community health workers [26,29,64]. Seven papers reported on setting up women’s groups as a means of providing peer counselling, community support, and increased prioritisation of women’s health issues [58,59,61,64-66]. Improvements were seen in clean delivery practices, early breastfeeding, improved nutrition during pregnancy and improved health care seeking for neonates [63,64]. Having high population coverage and high proportions of pregnant women participating were both significant predictors of effect [67].

### Exercise

Two RCTs assessed the effect of exercise during pregnancy in women who were overweight or had gestational diabetes mellitus (GDM) [68,69]. One found no reduction in preterm or stillbirth, but did find a significant reduction in GDM (22.0% vs 40.6%; *P* < 0.001) [68]. The other involved both an exercise regime and dietary changes and this was associated with reduction in preterm birth (OR = 1.64, 95% CI = 1.14-2.36) [69].

### Indoor air pollution

One RCT trialled a low-emission ethanol cook stove (as opposed to traditional kerosene stoves) to determine the differential effects of ethanol vs kerosene cook stoves on pregnancy outcomes [70]. There were no significant difference in rates of preterm birth or stillbirth, but there was a significant increase in extended perinatal mortality (7.9% vs 3.9% *P* = 0.045) (stillbirth or death within first 28 days of life) associated with kerosene cook stoves.

### Malaria prevention

Five trials involved malaria prevention during pregnancy [71-75]. Interventions included bed net provision plus indoor residual spraying (IRS), community scheduled screening and treatment plus intermittent preventative treatment (IPT) with Sulphadoxine Pyramethanine (SP) and all showed reductions in preterm or stillbirth. IRS led to decreases in neonatal mortality (17.2 vs 1.5% *P* = 0.006), stillbirth (7.5% vs 0% *P* = 0.03) and placental parasitaemia [71]. These reductions were seen despite poor compliance with the full course of SP – even with Community Health Worker (CHW) home delivery and Directly Observed Treatment Short-Course (DOTS), only 43% of women received the recommended 3 doses [72].

### Anti-helminthic therapy

One systematic literature review reported on anti-helminthic treatment, focusing specifically on community-based eradication programmes [76]. This found no significant difference in preterm birth or stillbirth reduction (RR = , 1.54 95% CI = : 0.93-2.58).

### Guideline implementation

Four studies evaluated the effect of guideline implementation for pregnancy care [18,77-79], and all had some beneficial effects on either preterm birth, stillbirth or neonatal mortality. The first study evaluated the impact of a guideline for the first ANC visit consisting of a checklist for health care workers. This led to a reduction in a composite outcome including stillbirth, preterm birth and low birthweight (RR = 0.72, 95% CI = 0.65-0.93) [18]. The second implemented a labour management guideline in the form of a pocket book ad wall posters, and observed a significant reduction in stillbirth (RR = 0.66, 95% CI = 0.53-0.82) [77]. Mgaya et al. found the introduction of a guideline for diagnosis and management of obstructed labour was followed by a reduction in perinatal mortality (stillbirth and neonatal mortality within the first 72 hours of life) from 16% to 8.8% (*P* = 0.01) [78]. Finally, Okonofua et al. found guidelines for diagnosis and management of eclampsia reduced the case fatality rate from 15.1 to 3.2% (*P* < 0.001) [79].

### Health systems strengthening

Five studies evaluated efforts to strengthen health systems [78,80–83]. A variety of methods were used though mainly included audit or quality improvement cycles where baseline data was collected and used to identify key areas of deficiency before prioritising and implementing intervention packages to address these [78,82]. In general, improvements were seen in outcome measures and this resulted from improvements in practice as well as more efficient and effective use of resources [82,83]. For example, a Ghanaian study trialling a series of QI bundles developed following a prolonged needs assessment, and observed a 36% reduction in stillbirth [83]. A Tanzanian study ran a series of audit cycles and achieved significant reductions in stillbirth and perinatal death [84], and a Zimbabwean study found a series of changes in leadership and accountability led to a reduction in intrapartum stillbirth to almost zero [82].

### Staff training

Nine implementation studies examined the impact of staff training on maternal and neonatal outcomes [18,61,78-81,83,85,86]. All studies reported some level of improvement in stillbirth, neonatal mortality or maternal mortality to varying degrees. Programmes involving repeated sessions reinforcing knowledge were more effective than one-off sessions [79,86]. Programmes were only effective if administrative authorities were involved from the outset [79,86]. Involving pregnant women in training and education was also found to improve outcomes in one study [85].

### Delivery care

There were eight intervention studies about delivery care including one systematic literature review [22,87-93]. Four studies compared facility with home delivery or delivery with traditional birth attendant [87,90,91,93]. All studies, including a systematic literature review [87] found that facility delivery conferred worse outcomes both for the neonate and the mother with significantly higher rates of stillbirth and maternal mortality. The systematic literature review evaluated data from 9 studies from sub-Saharan Africa including 47475 women, and found no significant change in perinatal mortality between home and facility delivery (OR 1.21, 95% CI = : 0.79-1.84) but did show a significant increase in maternal mortality [87]. Khanam et al. conducted a prospective cohort study in Bangladesh covering 24271 births and found a significant increase in perinatal mortality (OR = 2.4, 95% CI = 2.08-2.76) [93].

### Pharmacological interventions

There were ten trials of pharmacological interventions for the reduction of preterm and stillbirth. Three studies investigated the impact of antiretroviral therapy (ART) [94-96]. One found no significant difference in rates of preterm birth in mothers receiving ART [95] and the other two found ART significantly reduced preterm birth [94,96]. This effect was increased if ART was started before conception [94]. One study compared single dose antibiotic prophylaxis to a multi-day course for routine C-section and found there was no significant difference in stillbirth or neonatal mortality between the two [97]. Vaginal progesterone for tocolysis in twin pregnancies was not found to increase gestational age at delivery [98], and a comparison of IM progesterone and nifedipine for threatened preterm labour found no significant change in either time to delivery or rates of Neonatal Intensive Care Unit (NICU) admission [99].

### Periodontal disease management

One small randomised controlled study of antiseptic mouthwash provision along with dental education found no significant differences in preterm (OR = 1.59, 95% CI = 0.51-4.92) or stillbirth (OR = 1.01, 95% CI = 1.06-12.22) [100].

### Neonatal interventions

**Tables 7-12** show detailed characteristics of all included studies reporting on neonatal interventions.

### Feeding & nutrition

There were twenty-two studies of nutritional interventions in small or sick newborns to reduce neonatal mortality [101–123]. Three large-scale randomised controlled trials on preterm neonates found no beneficial impact on neonatal mortality from vitamin A supplementation, a result echoed by a subsequent meta-analysis including these studies [124].

A meta-analysis of three community studies in Ghana, Nepal, and India reported a significant reduction in all-cause neonatal mortality associated with initiation of breastfeeding within 24 hours compared with commencement later than 24 hours (OR = 0.56, 95% CI = 0.40-0.79) [109].

### Thermal regulation & homeostasis

The burden of neonatal hypothermia in babies born in hospital in LMICs has been estimated at 32%-85% and is an important contributor to preventable neonatal mortality, with one study reporting an 80% increase in adjust mortality for every degree Celsius drop in first observed body temperature [125,126]. Seven studies reported on thermal regulation [110,127-132]. A recent Cochrane review and meta-analysis of Kangaroo Mother Care (KMC) found in an LMIC subgroup analysis a reduction in all-cause neonatal mortality by 43% (OR = 0.57, 95% CI =  0.37-0.89), highlighting the significant potential impact of this low-cost intervention. One study reported a multi-faceted quality improvement project targeted at reducing transitional hypothermia in very low birthweight infants including implementing radiant warmers in the delivery room and specialised transport equipment along with comprehensive staff training. The initial results of this small study of 192 neonates showed a sustained improvement in normothermia (56% normothermic on arrival to NICU compared with 19% pre-intervention) and a significant decrease in mortality [128].

### Infection prevention & management

Thirty studies reported on this important aspect of neonatal care [102-106,109,111-115,117,122,123,130,133-147]. Topical emollient therapy was suggested in the data analysis for the Every Newborn series to have a significant impact on neonatal mortality [148], however, this has been challenged by subsequent research. A recent Cochrane review including 5 studies published in the last 5 years showed no significant reduction in neonatal mortality (RR = 0.94, 95% CI = 0.81-1.08) or incidence of invasive bacterial infection [149].

Probiotics and synbiotics have been studied for their role in preventing necrotizing enterocolitis (NEC) and associated mortality. A randomized controlled trial of 4000 newborns in India showed a 40% reduction in a combined endpoint of sepsis/death (RR = 0.60, 95% CI = 0.48-0.74) associated with once daily administration of the probiotic *Lactobacillus Reuteri* [103]. Importantly this study excluded neonates born before 35 weeks gestation or <2000g in birthweight however a meta-analysis of probiotics in preterm infants in LMICs also showed a significant reduction in all-cause mortality (RR = 0.73, 95% CI = 0.59-0.90), incidence of NEC (RR = 0.46, 95% CI = 0.34-0.61)and incidence of late-onset neonatal sepsis (RR = 0.80, 95% CI = 0.71-0.91) [102].

### Respiratory support

We found 25 studies exploring respiratory support in LMICs [150-173]. The implementation of bubble continuous positive airways pressure devices (bCPAP) has been shown to be feasible in multiple LMIC settings, in secondary and tertiary level facilities [151,153-156,160,163,166,170,171]. 2 recent systematic reviews have examined the impact of this on neonatal outcomes, specifically in LMICs [152,161]. Although there were no RCT data on mortality available, a pooled analysis of 4 observational studies showed bCPAP to have a major impact on mortality in preterm neonates (OR = 0.34, 95% CI = 0.14-0.82) [161]. Importantly, a study from a neonatal unit in Malawi showed that this beneficial effect is strongly mediated by neonatal temperature, with hypothermic neonates failing to respond to bCPAP [174], emphasising the importance of basic neonatal care as a requirement prior the addition of more complex interventions such as bCPAP.

Nine middle-income country studies were identified investigating the feasibility and impact of surfactant administration to premature neonates with neonatal respiratory distress syndrome (RDS) [150,151,158,159,162,167,169,172].Predominantly these were small-scale studies of either differing methods of surfactant administration or comparisons of early vs late administration, both of which showing inconsistent results. It appears that surfactant is a feasible intervention to implement in LMICS, and a meta-analysis showed reductions in mortality in eligible infants comparable with those observed in high-income settings (RR = 0.67, 95% CI = 0.57-0.79)[159,175]. Unfortunately, however, the high cost of the intervention and the infrastructure required in terms of delivery and ventilatory support is likely to leave surfactant beyond the reach of many low-income facilities for some time to come [159].

### Community mobilization

We identified 11 studies investigating aspects of community mobilization with regards to neonatal mortality reduction [25,59,63,107,131,176-181]. These largely focused on predominantly maternal interventions (eg, participatory women’s groups) and so are discussed above in the maternal interventions section.

### Cardiovascular support

There were two studies of interventions for cardiovascular therapy [182,183]. A Brazilian study of very low birthweight neonates with patent ductus arteriosus (PDA) showed a significant mortality reduction with pharmacological or surgical treatment of PDA compared with conservative management [182]. A Cochrane review reported low-quality evidence suggesting paracetamol to be as effective as indomethacin in PDA closure however highlighted insufficient follow-up data to establish evidence on neurodevelopmental outcomes of this treatment [183].

### Training programmes & resuscitation

Nineteen studies described implementation of training programmes [63,86,128,130,139,141,157,181,184-195]. Thirteen of these described training programmes in neonatal resuscitation, specifically the Helping Babies Breathe programme (HBB). Neonatal resuscitation was identified as a potentially very impactful intervention in the Every Newborn series. Studies investigating this subsequently have highlighted additional complexity in low-resource settings. A large study of HBB implementation in Nepal involving over 40 000 deliveries showed a significant reduction in intrapartum stillbirth and neonatal mortality in the 1st day of life however, no reduction in overall perinatal mortality (stillbirth or neonatal death within 7 days of life) [186,192].Other studies in India, Kenya and Tanzania, and 2 recent meta-analyses showed similar results [184,185,194-196]. These data suggest that although HBB training was effective in prolonging life of babies with intrapartum-related complications or those previously categorised as fresh stillbirths, the quality and availability of care in the postnatal period was not sufficient to sustain these gains [192]. In addition, although babies with severe asphyxia may have been successfully resuscitated, in reality the preferred intervention would have been earlier delivery by timely Caesarean-section. This highlights the importance of a systems-based approach to improving the continuum of care.

### Health systems strengthening & guideline implementation

Twenty studies were identified that described aspects of guideline implementation or health systems strengthening to improve outcomes of preterm babies in LMICs [59,63,104,128,130,135,142,144,145,157,177,178,180,181,188,191,197-200].

One initiative which targeted several different health system issues and showed impressive results was the development of a health partnership between 4 hospitals (2 university, 2 district) in Rwanda and a team of neonatologists and neonatal nurses from the United Kingdom [157]. They conducted a detailed needs assessment in all facilities and identified 6 major areas of need including nutrition, respiratory support, thermoregulation, and enhanced record keeping. Targeted intervention packages were developed for each of these needs, with examples being KMC utilisation for thermoregulation and training staff in breast feeding support and developing systems for storing expressed breast milk to improve nutrition. The total budget of the project was only US$45 000 and yet overall in-hospital neonatal mortality decreased from 23.6% to 21.7% in university hospitals and from 10% to 8.1% in district hospitals (*P* = 0.056).

On a smaller scale Harris et al reported results from a pre- post-implementation trial aiming to improving neonatal outcomes in a single Ugandan neonatal unit [198]. Protocols for management of unwell newborns were developed using literature and local consensus and disseminated through ward meetings and regular ward-based teaching. Audits at 3 and 6 years showed a reduction of neonatal mortality from 0.58 deaths per admission to 0.26 and 0.17 deaths respectively. The sustained nature of this improvement without major investment in infrastructure is particularly striking.

## DISCUSSION

This review has highlighted a set of measures with a strong evidence base and the potential to prevent a large proportion of stillbirth and preterm birth, and reduce neonatal mortality in low- and middle- income settings. Maternal interventions found to be successful were antenatal care, multiple micronutrient supplementation and malaria prevention. Successful neonatal interventions included vitamin A supplementation, probiotics and early breastfeeding initiation, KMC and bCPAP. Staff training and guideline implementation and community mobilisation were beneficial for both mothers and neonates. Many of these interventions are relatively simple and it is clear that new innovations are not a priority- we need innovative ways to successfully implement the interventions we know can work.

### Strengths and limitations

Our study has several important strengths. Our explicit focus on LMIC evidence delivered an important set of conclusions with direct applicability to LMIC policy-makers, something that is increasingly crucial in the context of the ACT trial and other recent findings. By intentionally limiting our review to the last 5 years we were able to focus on changes to the evidence base since the Every Newborn series and highlight important interventions with new recommendations or that merit further investigation such as optimising neonatal thermal care, and minimising indoor air pollution. The production of evidence gap maps is a relatively novel interactive way of presenting this visually and can play an important role going forward in understanding the current state of evidence for maternal and newborn health interventions. By investigating maternal and newborn interventions in conjunction we were able to draw important conclusions regarding the continuum of care involved and the role of whole-system approaches to improving outcomes.

There are also several limitations to consider. First, although the review was intentionally limited to the last 5 years to focus on changes to the evidence base since the Every Newborn series, this could have resulted in missing important evidence generated prior to 2013 but not included in the Every Newborn publications however we feel this is unlikely. We only included maternal interventions delivered to pregnant women, thereby excluding other population-level interventions delivered to all women of childbearing age which may have an impact on preterm birth and stillbirth incidence (eg, access to birth control) [201,202]. LMICs are a broad and heterogeneous group and some of our conclusions may not be transferable to LMIC contexts other than those where the studies took place.

### Strategies for stillbirth reduction

Many stillbirths can be prevented by relatively simple measures. Facilitating condition recognition and diagnosis of high-risk pregnancy is the cornerstone and this can in large part be achieved by enabling early antenatal care attendance, providing appropriate staff training and guidelines, and ensuring women are able to access the health care they require. Additionally, the ability to perform timely delivery is crucial- this means the ability to induce labour or perform emergency Caesarean-section without undue delay caused by lack of facilities, limited staffing, or infrastructure (eg, transport) problems.

Access to quality antenatal care is a key priority and has been clearly demonstrated to reduce perinatal mortality [17,20,22,26,203]. Data from Ghana showed that receiving high-quality antenatal care halved risk of stillbirth as it enabled provision of malarial treatment and prophylaxis, screening for anaemia, helminth management and blood pressure monitoring. In addition it facilitated Prevention of Mother to Child Transmission of HIV (PMTCT) and syphilis detection and treatment. It also allowed provision of nutritional supplements and an opportunity for educating women about ways to improve their health and recognition of danger signs in pregnancy [17]. Modelling has predicted that the provision of 10 basic services in antenatal care could avert 45% of all stillbirths [204]. As part of strategies to reduce both perinatal and maternal mortality, there has been increasing efforts in many LMICs to encourage women to give birth in health facilities in order to allow early complication recognition and life-saving interventions. However, in many settings facility based delivery has been associated with paradoxically worse outcomes compared with home delivery [22,87,90,93]. This is multifactorial, and in part can be attributed to increased likelihood of high-risk cases being selected for facility delivery. In Ethiopia, a cohort study of 4442 women found that women who experienced intrapartum complications were twice as likely to deliver in a health facility than those who did not, explaining in part the fact there was no stillbirth reduction in facility births [22]. However, this is not the only modifying factor and quality of obstetric care available is likely to be a key determinant. One study using Demographic Health Survey (DHS) data from Malawi for example found that, even when controlling for the risk profile of patients, facility delivery conferred poorer outcomes for both the mother and the baby [90]. The additional risk burden is likely to be attributable to the fact that increasing uptake of facility delivery does not translate into increasing availability of high quality CEmONC care. This has been demonstrated in multiple low- and middle-income settings where, even when women are able to reach health facilities, access to the care they need is limited by lack of medical supplies such as blood, or lack of staff with sufficient expertise and training [205]. This highlights the need for coordinated systems and infrastructure development in conjunction with service provision in order to provide the necessary care.

### Strategies for preterm birth reduction

Preterm birth reduction is complex and involves national level commitment to improving maternal health and well-being. Specific interventions with a strong evidence base in LMICs include optimising nutritional intake, which is important for both micro- and macro-nutrients, and there is a wealth of evidence for different nutritional supplements which reduce preterm birth rates [5,36,42,43,46,47,55,56,206]. Appropriate HIV management is also important [96],as is adequate malarial prophylaxis [71,73,75].

Strategies to improve women’s health at a population level however must be in tandem with advocacy for women’s rights. Intimate partner violence is a threat to women’s well-being worldwide and lack of female empowerment means many women are unable to make safe decisions about their reproductive health. Improving access to contraception, family planning, and abortion is crucial in forwarding female- and therefore maternal health.

### Strategies for neonatal mortality reduction

Our findings have shown a number of interventions which can reduce neonatal mortality. Early initiation of breastfeeding, KMC and probiotics are all simple, low-cost interventions which could be easily implemented at low cost. There are also promising results from use of bCPAP and surfactant. Introduction of HBB training showed promise in mortality reduction however also served to highlight the need for systems strengthening alongside HCW training.

Kanagroo Mother Care (KMC) to aid thermal regulation in babies <2000g birthweight has been rolled out across LMICS as part of the Every Newborn Action Plan [207]. Currently in the majority of settings KMC is initiated only on stable neonates once they have completed any required treatment and so unstable babies on treatment remain at high risk of hypothermia. The results are awaited of an ongoing WHO multi-site LMIC trial of immediate KMC (i-KMC) where KMC is initiated immediately after birth regardless of other ongoing treatments [208] and may provide an important recommendation of the benefit of KMC even in unstable babies in the first days of life.

This review has also shown important changes to the evidence base subsequent to the Every Newborn Action Plan series of reviews and emphasised the importance for LMIC health policy development of considering evidence derived in LMICs. We have derived important conclusions regarding general and intervention-specific barriers and enabling factors to implementation, which are essential to consider when developing new maternal and newborn health policy initiatives.

### Implementation challenges

Our review has identified that while much of the burden of stillbirth and preterm birth can be prevented by interventions already available, the real challenge is in the successful implementation of these strategies. A number of common themes arose in our review highlighting some of the key areas which must be addressed in order to create successful strategies.

#### Health systems strengthening

Many of the deficits in health care in LMICs are due to weak health care systems and a lack of robust protocols and support systems. These are often deeply ingrained, multi-faceted and challenging to address, however without such attempts, only limited improvement in outcomes can be achieved.

Quality improvement projects are a burgeoning area of research in LMICs as they require relatively low resource input and can effectively target areas of need. Success is however dependant on investment of facility staff in the cycle and in the ongoing re-evaluation of outcome measures [83]. Some successful attempts to improve this have included use of “local champions” and also leadership boards allowing inter-site comparisons [83]. Several studies delivered and evaluated HBB through QI methodological approaches. The value of these were apparent, one example being the utilisation of intermediate data in the form of ‘run charts’ to highlight the impact of staff turnover on outcomes and supplement advocacy at hospital administration level [209]. In an era of renewed global focus on quality of care, pragmatic trials and learning health systems, these approaches are likely to prove highly important [210-212].

One initiative which targeted several different health system issues and showed impressive results was the development of a health partnership between 4 hospitals (2 university, 2 district) in Rwanda and a team of neonatologists and neonatal nurses from the United Kingdom [157]. They conducted a detailed needs assessment in all facilities and identified 6 major areas of need. Targeted intervention packages were developed for each of these needs, with examples being KMC utilisation for thermoregulation and training staff in breast feeding support and developing systems for storing expressed breast milk to improve nutrition. The total budget of the project was only US$45 000 and yet overall in-hospital neonatal mortality decreased from 23.6% to 21.7% in university hospitals and from 10% to 8.1% in district hospitals (*P* = 0.056). The project emphasized the potential for locally-driven health systems strengthening through partnership alongside government support and also highlighted the important barrier to training of high staff turnover which hampered the development of institutional memory with regards to new working practices.

#### Guideline implementation

In many LMICs, the majority of health care is not performed by doctors but by clinical officers and other lower skilled health care workers. There has been much success in introducing algorithmic approaches to health care, allowing lower skilled workers with less expertise to assess and manage sick patients, however for guidelines to be successful, they must be implementable and accessible to the staff who use them. Relevant care providers should be identified and involved in establishing standards and developing guidelines and protocols which are locally relevant. This ensures guidelines are applicable, attainable, and gives clinicians ownership over them, meaning they are more likely to be adhered to [84].

The most successful models were those combining guidelines with training programs and education, and much greater levels of adherence were achieved [18,79,84]. This was augmented further with the use of audit cycles to evaluate the guidelines and improve on them and their usage over time [84]. Ultimately Guidelines will only lead to improved quality of care if they are used in daily clinical practice therefore staff need to be supported in order for this to happen with reminders, monitoring and feedback [18].

#### Staff training

Staff training is imperative in achieving optimal maternal and neonatal care. A systematic literature review evaluating perinatal mortality found that across 9 centres in Sub-Saharan Africa, perinatal mortality was 21% in facilities than with home deliveries [87]. Although some of this may be because more complex pregnancies are more likely to attend facilities for delivery, much of this is due to the inability of staff to appropriately manage complications. In Ethiopia for example, only 1.3% of facilities were able to provide Basic Emergency Obstetric and Neonatal Care (BEmONC) [22].

One-off staff training programmes rarely produced successful outcomes and there is a need for repetition of training allowing reinforcement of new skills and knowledge over time [79,86]. Training should take place in the context of regular audits of outcomes allowing identification of successes and also of failures to feedback to staff allowing continued development of knowledge and skills [79].

It is crucial to involve authorities such as Ministry of Health or hospital administrative staff from an early stage to ensure the staff are supported and encouraged to attend training sessions, and also to adopt new practices. Administrative bodies must also be involved to enable the supply of the necessary equipment to implement new skills and practice [79,86].

Necessary staff must be identified and enabled to attend the training session. In health facilities, a critical mass of training staff is needed in order to effect systems change [86] · This is particularly challenging to achieve in settings where staff retention is poor and turnover is high, as is frequently the case in low resource health facilities [214].Strategies need to be put in place to incentivise staff retention or facilitate frequent training provision and again this will mandate involving administrative staff and relevant officials from the outset [82].

As well as improving the skillset of staff, it is important to involve women in the outcomes of their pregnancy and to empower them with increased skills and advocacy to effect change both on their own outcomes, but also the wider health services in their community [85]. When trying to improve maternal and child health, this must take place in parallel with increasing the voice and the rights of women, and education is the cornerstone of this approach.

#### Community Groups

Community groups and community mobilisation are examples of relatively low-cost, low-resource, low-intensity innovations with the potential for significant effect. There are various models however they are mainly based around participatory learning and action cycles run within small groups of women led by a trained (and usually salaried) facilitator [65]. This model enables identification and prioritisation of problems, planning and implementation of strategies which are locally feasible, and re-assessment and evaluation [65].

There are a number of reasons why groups such as these can be so effective. They require minimal resources and empower communities to address locally relevant health determinants. They facilitate capacity building at a community level but also enable increased advocacy for local issues [178]. It is worth mentioning however, a large RCT performed as part of The Global Network study which implemented a package of interventions including community mobilisation and also health centre quality improvement and staff training. Despite being rolled out across five countries with considerable funding and ongoing support, they saw no improvement in their outcome measures over a two year period [61]. There are a number of possible explanations for this however it highlights the fact that, as with many of the interventions discussed in this review, community mobilisation can only have a significant impact on outcomes if it occurs alongside improvements in the abilities of the health service to manage obstetric emergencies effectively and in a timely manner.

### Research as part of routine care

Much of the emerging LMIC data highlighted in this review emphasizes the need for whole-system approaches to delivering individual interventions, and the concept of creating ‘learning health systems’ (LHS) has much to offer in delivering this and driving health system improvement in LMICs. In an LHS, data capture processes are optimised and embedded into routine care, clinical data entered once can then be repurposed many times for administration, quality improvement and research [214]. Although the technological and human resource infrastructure required to implement LHS are often lacking in LMIC contexts [215], there are important examples of where this has already been shown to be possible, such as the Clinical Information Network of paediatric departments in Kenya [210]. In addition the current lack of infrastructure offers the opportunity to implement LHS now, rather than retrofit them as has been necessary and very costly in many high-income contexts [216] · The potential for using LHS to conduct rapid pragmatic trials at low cost is particularly appealing in LMICs given the lack of evidence base for many intervention in these settings, and can support ongoing developments in LMIC quality improvement methodology as described above. LMICs stand potentially to benefit the most from learning health systems as a means of unifying fragmented approaches and producing systems capable of continuous improvement.

## CONCLUSION

Using intervention evidence generated in LMICs, this review has highlighted several areas regarding preterm birth and stillbirth reduction, and the management of small and ill newborns, where the evidence base has changed significantly since the Every Newborn Action Plan. The key point to emphasise is the potential to deliver marked reductions in preterm birth and stillbirth with relatively simple interventions, however these must be delivered as part of an approach of a whole-system strengthening to be effective. Learning health systems can offer an opportunity to bring the current fragmented context in many LMIC health systems together and provide important means of understanding implementation challenges and running rapid pragmatic trials, delivering locally relevant data at a low cost and driving continual improvement in quality of care.

## Additional material


Online Supplementary Document
