# Supplementary material for: Interventions to reduce preterm birth and stillbirth, and improve outcomes for babies born preterm in low- and middle-income countries: A systematic review
Source: J Glob Health. 2021 Dec 30;11:04050. doi: 10.7189/jogh.11.04050 (PMC8709903; doi:10.7189/jogh.11.04050)
Supplement: Online Supplementary Document [file jogh-11-04050-s001.pdf]

Table S1. Example search strategy*

**1**	premature birth.mp. or exp premature birth/
**2**	exp infant premature/
**3**	((pre-term or preterm or premature) adj1 (bab* or infant)).mp. [mp=title, abstract, original title, name of substance word, subject heading word, keyword heading word, protocol supplementary concept word, rare disease supplementary concept word, unique identifier, synonyms]
**4**	stillbirth.mp. or exp stillbirth/
**5**	fetal death.mp. or exp fetal death/
**6**	pregnancy complications.mp. or exp pregnancy complications/
**7**	pregnancy outcome.mp. or exp pregnancy outcome/
**8**	1 or 2 or 3 or 4 or 5 or 6 or 7
**9**	Epidemiologic studies/
**10**	exp case control studies/
**11**	exp cohort studies/
**12**	Case control.tw.

**13**	(cohort adj (study or studies)).tw.
**14**	Cohort analy$.tw.
**15**	(Follow up adj (study or studies)).tw.
**16**	(observational adj (study or studies)).tw.
**17**	Longitudinal.tw.
**18**	Retrospective.tw.
**19**	Cross sectional.tw.
**20**	Cross-sectional studies/
**21**	9 or 10 or 11 or 12 or 13 or 14 or 15 or 16 or 17 or 18 or 19 or 20
**22**	Randomized Controlled Trials as Topic/
**23**	randomized controlled trial/
**24**	Random Allocation/
**25**	Double Blind Method/

**26**	Single Blind Method/
**27**	clinical trial/
**28**	clinical trial, phase i.pt.
**29**	clinical trial, phase ii.pt.
**30**	clinical trial, phase iii.pt.
**31**	clinical trial, phase iv.pt.
**32**	controlled clinical trial.pt.
**33**	randomized controlled trial.pt.
**34**	multicenter study.pt.
**35**	clinical trial.pt.
**36**	exp Clinical Trials as topic/
**37**	22 or 23 or 24 or 25 or 26 or 27 or 28 or 29 or 30 or 31 or 32 or 33 or 34 or 35 or 36

**38**	(clinical adj trial$).tw.
**39**	((singl$ or doubl$ or treb$ or tripl$) adj (blind$3 or mask$3)).tw.
**40**	PLACEBOS/
**41**	placebo$.tw.
**42**	randomly allocated.tw.
**43**	(allocated adj2 random$).tw.
**44**	38 or 39 or 40 or 41 or 42 or 43
**45**	intervention*.mp.
**46**	Meta-Analysis as Topic/
**47**	meta analy$.tw.
**48**	metaanaly$.tw.
**49**	Meta-Analysis/
**50**	(systematic adj (review$1 or overview$1)).tw.

**51**	exp Review Literature as Topic/
**52**	46 or 47 or 48 or 49 or 50 or 51
**53**	reference list$.ab.
**54**	bibliograph$.ab.
**55**	hand-search$.ab.
**56**	relevant journals.ab.
**57**	manual search$.ab.
**58**	53 or 54 or 55 or 56 or 57
**59**	selection criteria.ab.
**60**	data extraction.ab.
**61**	59 or 60
**62**	Review/

**63**	61 and 62
**64**	Comment/
**65**	Letter/
**66**	Editorial/
**67**	64 or 65 or 66
**68**	52 and 58 and 63
**69**	21 or 37 or 44 or 45 or 68
**70**	69 not 67
**71**	8 and 70
**72**	(algeria or egypt or libya or morocco or tunisia or cameroon or central african republic or chad or congo or "democratic republic of the congo" or gabon or burundi or djibouti or eritrea or ethiopia or kenya or rwanda or somalia or sudan or south sudan or tanzania or uganda or angola or botswana or lesotho or malawi or mozambique or namibia or south africa or swaziland or zambia or zimbabwe or benin or burkina faso or "cape verde" or "cabo verde" or "cote d'ivoire" or "ivory coast" or gambia or ghana or guinea or guinea-bissau or "equatorial guinea" or liberia or mali or mauritania or niger or nigeria or senegal or sierra leone or togo or cuba or dominica or dominican republic or grenada or haiti or jamaica or "saint lucia" or "st. lucia" or "saint vincent and the grenadinesor st. Vincent and the grenadines" or belize or costa rica or el salvador or guatemala or honduras or nicaragua or panama or mexico or argentina or

	bolivia or brazil or colombia or ecuador or guyana or paraguay or peru or suriname or venezuela or antarctic* or arctic or kazakhstan or kyrgyzstan or kyrgyz republic or turkmenistan or tajikistan or uzbekistan or cambodia or east timor or timor-leste indonesia or laos or lao or malaysia or myanmar or philippines or thailand or vietnam or Bangladesh or bhutan or india or afghanistan or iran or iraq or jordan or lebanon or syria* or turkey or yemen or nepal or pakistan or sri lanka or china or tibet or korea or "democratic people's republic of korea" or mongolia or albania or bosnia herzegovina or bulgaria or belarus or macedonia or moldova or montenegro or russia* or serbia or ukraine or armenia or azerbaijan or georgia or fiji or papua new guinea or vanuatu or micronesia or samoa or "independent state of samoa" or american samoa or tonga or comoros or madagascar or mauritius or "solomon islands" or kiribati or kosovo or croatia or romania or "marshall islands" or sao tome principe or maldives or tuvalu or nauru or west bank or gaza or palestin* or "high-burden" or burden or countdown or "low-income" or "low income" or "middle-income" or "middle income" or "low-resource" or "low resource" or developing).mp. or Developing Countries/
**73**	71 and 72
**74**	Animal/ not Human/
**75**	73 not 74
**76**	limit 75 to yr="2013-current"

*Searches for maternal interventions used variations of the terms shown below for the search on MEDLINE database. Preterm birth and stillbirth terms were combined with Scottish Intercollegiate Guidelines Network search filters for different study types (https://www.sign.ac.uk/search-filters.html) and filters for low- and middle-income countries.
